# 

**Published:** 1916-05

**Authors:** 


### CONTENTS.

ORIGINAL COMMUNICATIONS—

Present Status of Pneumococcus Infections. By W. W. Young, A. B., M. B., Atlanta, Ga. ---- 1

Antiseptic Action of Hypochlorous Acid and Sodium Hypochlorite. By M. C. Pruitt, L. R. C. P., L. R. S. P., L. R. F. P. S., M. D. ---------- 7

Intravenous Treatment of Acute Articular Rheumatism. By Dr. T. C. Davidson, Atlanta, Ga. 12

Regular Meeting of the Fulton County Medical Society, Thursday, April 6, 1916 ---------- 14

EDITORIAL ---------- 18

SELECTIONS AND ABSTRACTS ---------- 20

BOOK REVIEWS ---------- 43

